# Development of a novel bivalent vaccine candidate against hepatitis A virus and rotavirus using reverse vaccinology and immunoinformatics

**DOI:** 10.1016/j.jve.2024.100578

**Published:** 2025-01-23

**Authors:** Hassan Yarmohammadi, Abbas Akhavan Sepahi, Mojtaba Hamidi-fard, Mohammadreza Aghasadeghi, Golnaz Bahramali

**Affiliations:** a Department of Microbiology, Islamic Azad University, North Tehran Branch, Tehran, Iran; b Department of Hepatitis and AIDS, Pasteur Institute of Iran, Tehran, Iran

**Keywords:** Recombinant vaccine, Immunoinformatic, Rotavirus, Hepatitis a virus, Bivalent vaccine

## Abstract

The hepatitis A virus (HAV) and rotavirus are mainly transmitted through fecal-oral and person-to-person contact, and cause severe gastrointestinal complications and liver disease. This work used reverse vaccinology and immunoinformatic methods to create a novel bivalent vaccine against rotavirus and HAV. The amino acid sequences of HAV-rotavirus proteins (VP1 and VP8∗) were retrieved from the GenBank database. Various computational approaches were employed to predict highly conserved regions and the most immunogenic B-cell and T-cell epitopes of VP8 and VP1 of rotavirus and HAV proteins in both humans and BALB/c. Moreover, the predicted fusion protein was analyzed regarding primary and secondary structures and homology validation. In this study, we used two highly conserved peptide sequences of VP8 and VP1 of rotavirus and HAV that induce T and B cell immunogenicity. According to T-cell epitope prediction, this area comprises 2713 antigenic peptides for HLA class II and 30 HLA class I antigenic peptides, both of which are virtually entirely conserved in the Iranian population. In this study, validation as well as analysis of the secondary and three-dimensional structure of the VP8∗-rotavirus + AAY + HAV-VP1 fusion protein, with the aim of designing a multi-epitope vaccine with different receptors. TLR 3, 4 high immunogenic binding ability with immunological properties and interaction between multi-epitope target and TLR were predicted, and it is expected that the target fusion protein has stable antigenic potency and compatible half-life. The above is suggested as a universal vaccination program.

## Graphical abstract

Schematic view of the applied methodology has been illustrated.

**Figure:**
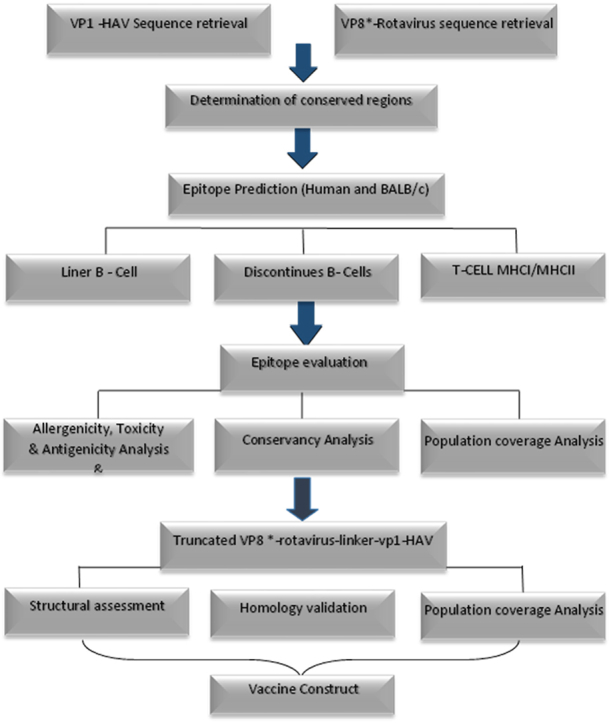

## 1 Introduction

Various human viruses can multiply in the digestive system and the most common way of their transmission is through the fecal-oral route, water and food contamination, as well as a result of the indiscriminate discharge and leakage of sewage in the living environment and absence of treatment, high-risk human behaviors such as abandoning feces of sick people in play areas and work environment, which can contaminate running water and even penetrate into tap water sources, are among the factors helping the spread of intestinal viruses. and facilitating the cycle of further transmission to vulnerable people. The most common viruses that are spread in this way are rotaviruses and HAV, which are responsible for acute hepatitis and gastroenteritis, respectively.^1^^,^^2^

HAV, a member of the Hepatovirus genus generally causing a self-limiting illness, belongs to the Picornaviridae family. The World Health Organization (WHO) estimates that yearly there are bout 30,000 and 35,000 fatalities and 100 million HAV infections. Several nations in the Middle East and Northern Africa (MENA) area, including Iran, have high or extremely high HAV endemicity levels. Recent research indicates that during the past few decades, the frequency of HAV infection in Iran has dramatically reduced. However, the virus is still widespread in some portions of the nation, notably in border regions and rural areas with poor sanitation. Adults with lower socioeconomic level have higher immunogenicity and more asymptomatic HAV infection, and vice versa.^1^

The Reoviridae family of viruses, which includes rotaviruses, is divided into seven groups (A-G). In developing nations in Africa, Asia, and Latin America, it is estimated that there are 744 million to 1 billion cases of diarrhea and 2.4 to 3.3 million fatalities among children under the age of five per year. This equates to 6600 to 9000 deaths every day. The most common cause of severe gastroenteritis in children under five years of age in developing and industrialized and less developed countries are rotaviruses.^3^

HAV and rotavirus immunisations are now accessible. There are presently two approved oral rotavirus vaccines on the market^2^^,^^4^: RotaTeq® from Merck^5^ and Rotarix® from GlaxoSmithKline.^6^ There are three approved HAV vaccines, including the inactivated combination vaccine Twinrix and single-antigen vaccines Havrix and Vaqta.^7^^,^^8^ The safety and effectiveness of all three using inactivated HAV, are equivalent and available in the USA. However not everybody, especially in low-income countries, has access to them.^9^ This emphasizes how urgently more work has to be done to guarantee that these vaccines are available and inexpensive for all populations, regardless of their financial situation.^5^^,^^10^^,^^11^

The VP1, VP2, VP3, and VP4 capsid proteins are all present in 60 copies in the HAV capsid.^12^ The interaction between the exterior surface of the constructed capsid and a receptor on the surface of the host cell is necessary for the virus to enter the cell. The capsid surface also interacts with the host's antibodies, which may be able to neutralize the virus and stop infection. 42 antigenic areas of the HAV polyprotein were discovered using peptide mapping in earlier investigations.^13^ Numerous investigations have demonstrated that VP1 stimulates the development of antibodies that are neutralizing.^14^ As a result, it is thought that the VP1 protein may be a candidate for a recombinant vaccination.^15^ Rotavirus, on the other hand, has a segmented genome that codes for six structural and six nonstructural proteins.^16^ The outer surfaces of the virions include the two key structural proteins VP4 and VP7.^17^ By way of proteolytic cleavage, VP4 is split into two subunits, VP5 and VP8.^18^^,^^19^ It is thought that VP8∗ plays a key role in viral attachment to host cells.^20^, ^21^, ^22^

The study of peptide-based vaccinations has grown in popularity. Long peptides with immunodominant epitopes may be employed as vaccines if they can stimulate both humoral and cellular immunity against a variety of serological types.^23^^,^^24^ The terms “multivalent” and “multivalent vaccine” are frequently used imprecisely to refer to two distinct vaccine types^25^: one that may provide protection against numerous illnesses with a single vaccine and one that can provide protection against multiple strains of a single pathogen.^25^, ^26^, ^27^ Because they can protect against several illnesses with fewer injections, lower the cost of healthcare and patient care, and streamline vaccination programs while preserving the effectiveness and safety of single-component vaccines, efficient multivalent vaccines are highly desired.^28^^,^^29^ It is vitally necessary to develop a multivalent peptide-based vaccination against HAV and rotavirus, especially in low-income nations where the illness burden is high and access to clean water and healthcare is constrained.^30^^,^^31^

Bioinformatics tools of multi-domain analysis, essential estimation in fusion protein production to elucidate immunologically conserved residues, lead to the emergence of new disciplines in the field of immunoinformatics-based multi-epitope vaccine design and construction,^32^ such as epitope prediction, efficacy analysis, safety, it provides the effects of toxicity without the need for the growth of pathogenic agents and thus accelerates the vaccine development process.^25^^,^^33^

The goal of this work is to create a new, recombinant VP8∗-rotavirus + AAY + HAV-VP1 fusion protein utilizing a flexible linker with a proteasome-cleavable site. This protein combines VP4 (VP8∗) with the immunodominant portions of VP1. This strategy is to develop a bivalent vaccination candidate that may concurrently protect against rotavirus and the hepatitis A virus.

Success of such a vaccine would depend to a large extent on the antigenic peptide to be used in antibody production. The non-glycosylated protein VP4 on the surface capsid of the virus is important in Rota viral immunogenicity and the major antigenic site(s) responsible for neutralization of the virus via VP4 is in the VP8∗ subunit of VP4. It is necessary that the peptide should be very specific and a peptide sequence which would stimulate both the T and B immunogenic cells would provide maximum protection against the virus. Advanced computational techniques and existing databases of sequences of the VP4 protein of rotavirus help in identification of such specific sequences. Using an Insilco approach, we have identified a highly conserved VP8∗ subunit of the VP4 surface protein of rotavirus which shows both T and B cell processivity and is also non-allergenic. This sub-unit could be used in *in vivo* models for induction of antibodies^19^ G1P8 rotavirus strain As the dominant strain responsible for the spread of rotavirus infection in Iran and other parts of the world, we consider it in this study.

## 2 Material and methods

### 2.1 Amino acid sequence retrieval

The VP1-HAV (NP_740552.1), VP8∗ rotavirus G1P^8^ ALU63988.1), CAC14074.3 (IIIA), AAU87586.1 (IIA), BAF37541.1 (IIIB), AAK44219.2 (IIB), and AF314208.1 (IB) sequences were obtained in FASTA format from GenBank of the National Centre for Biotechnology Information (NCBI).^34^

### 2.2 Determination of conserved regions

The obtained sequences were aligned using MEGA6.0 ClustalW and the multiple sequence alignment (MSA) program to determine the degree of conservation. Using the Bio edit tool, MSA was visualized.^35^^,^^36^

### 2.3 Prediction of T-cell epitopes in BALB/c and human

We used a combined peptide library to evaluate and evaluate matrices (Comblib Sidney2008) and submitted the acquired sequences to the IEDB's MHCI- and MHCII-binding prediction tool (http://tools.iedb.org/mhc/n) using a variety of techniques, including artificial neural networks (ANN), stabilized matrix methods (SMM), and SMM. Additionally incorporated were Rank PEP server and MHC-NP net CTLpan1.1 server. Results from all employed procedures all came within a similar range.

T-cell epitope lengths were set at 9 mers for MHC class I and 15 mers for MHC class II for BALB/c and human, respectively. The MHC class I alleles for BALB/c were H2-Dd, H2-Kd, and H2-Ld, whereas the MHC class II alleles were H2-IAd and H2-IEd. Based on the diversity of antigens and the level of identification by the variable HLA molecules in a population, as well as the most prevalent HLA in the Persian population based on the available report, HLA-A∗01, 02, 03, 11, 24, 26, 32, HLA-B∗35, 51, 50, 27, 57, and HLA-DRB1∗15, 11, 13, 03, 04, 07 were selected for MHC class I and MHC class II, respectively.

### 2.4 Predictions of antigenicity, immunogenicity, population coverage, and epitope conservancy

The antigenicity scores of discovered epitopes were assessed using the online VaxiJen v2.0 antigen prediction programme,^37^^,^^38^ which enables the arrangement of antigens according to the physicochemical properties of the protein without the need for sequence alignment. Antigenic scores greater than 0.5 4 were used to define such epitopes. Through the IEDB tool in the Iranian population (http://tools.iedb.org/population/), the predicted peptides interacting with MHC I and II molecules in the considered area were investigated.^39^

### 2.5 Toxicity and allergenicity analyses

We evaluated the toxicity of the selected model using ToxinPred.^40^ This tool may be used to check all physicochemical properties to confirm that epitopes are safe for the host. The allergenicity was examined using AllerTOP v.2.0.^41^

### 2.6 Prediction of linear and conformational B-cell epitopes

The BepiPred^42^ linear epitope prediction service from the Immune Epitope Database was used to forecast linear B cell epitopes with a threshold of 0.35 and lengths ranging from 6 to higher residues. The approaches for identifying various physicochemical characteristics of amino acids, such as antigenicity, surface accessibility, flexibility, hydrophilicity, and beta-turns, were also evaluated using the tools of the Immune Epitope Database (IEDB) Analysis Resource (http://tools.iedb.org/bcell) platform. The protein sequence scanning window was extended in all approaches to 389 residues. With a minimum score of 0.50, we utilized the IEDB online programme ElliPro to predict discontinuous B-cell epitopes. This technique predicts epitopes by taking into account both structure- and sequence-based information.

### 2.7 Primary and secondary structure assessment

The physicochemical characteristics of the fragments, including their weight, aliphatic index, Grand average of hydropathicity (GRAVY), theoretical pI, and atomic composition, were examined using Expasy's ProtParam service. The self-optimized prediction method with alignment (SOPMA) and Jpred tools were used to assess the secondary structure of the proteins' a-helix, b sheets, and random coils.^43^

### 2.8 Homology modeling and validation

The Threading ASSEmbly Refinement (I-TASSER) online server programmes were developed by Zhang Y. (2008). Protein 3D Structure Prediction I-Tasser Server. IntFOLD Integrated Protein Structure and Function Prediction Server,^41^ which provides 3D models as well as a confidence score (C-Score) and a model quality score, was used in the analysis of the 3D models (BMC Bioinformatics). Then, an analysis of the three DFIRE2 indicators—energy profile, C-score, and stereochemical qualities—was conducted.^40^^,^^44^ Utilizing the GalaxyRefine server (http://galaxy.seoklab.org/), the projected vaccine build model will be improved and refined.^45^ The ProSA-web server was used to evaluate the three-dimensional model^46^ (https://prosa.services.came.sbg.ac.at/prosa.php). Additionally, the Ramachandran graph of the three-dimensional structure was created using the MolProbity service (http://molprobity.biochem.duke.edu/).^47^

### 2.9 Molecular docking analysis

To activate innate immune cells in antiviral responses, Toll-like receptors (TLRs) are used as integral membrane proteins on innate immune effector cells. TLR3 and TLR4 can recognize structural glycoproteins and specific forms of viral nucleic acids and nonstructural proteins, leading to the release of inflammatory cytokines, and are important in recognizing viral genetic material in end lysosomal compartments to initiate antiviral responses.^48^ To understand the interaction between their structure and TLRs, we obtained the crystal structures of TLR3 and TLR4 from the Protein Data Bank using PDB IDs 3FXI and 1Z1W. Subsequently, we used the ClusPro 2.0 server, which is based on the PIPER Fast Fourier Transform (FFT) correlation method, to bind TLR3 and TLR4 to vaccine protein as ligands for binary interaction. The docking process obtained 30 models through three sequential steps: rigid-body docking, cluster-preserving structures, and refinement using CHARMM minimization. The best cluster was selected based on the lowest score. Finally, PyMOL was used to visualize binding structures and evaluate interactions between TLRs and vaccines.^49^^,^^50^

## 3 Results

**Fig. 1:**
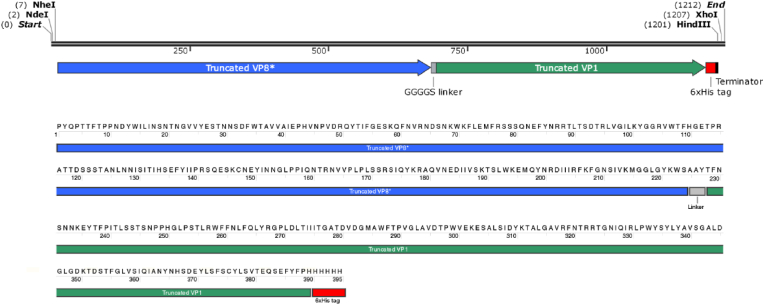
Schematics of the fusion protein construct.

The Iranian population's highly preserved VP8∗-rotavirus + AAY + HAV-VP1 fusion protein areas were chosen. The variable sections were removed in order to produce the most appropriate shortened version of HAV, and as a result, amino acids 99 to 259 in the VP1 alignment comprised the most conserved amino acids throughout the clades. Additionally, the viral VP8∗ amino acids 68 to 277 were used. This study led to the selection of VP8∗-rotavirus + AAY + HAV-VP1 fusion protein for further evaluation (Fig. 1).

### 3.1 MHC class I and II binding prediction in BALB/c and human

To design a more efficient and high-throughput vaccination model, we looked at the 9 m coverage of T-cell epitopes in BALB/c. (Table 1). According to the IEDB recommendations, the MHC-NP, net CTLpan1.1, RANKPEP, and netMHCpan3.0 servers were utilized to anticipate the potential epitopes. T-cell epitopes were evaluated using a variety of methods to improve prediction precision. High affinity peptides with the antigenic characteristic (percentile rank 1) are indicated as a result of the findings in Table 1. Accordingly, of the 42 MHC class I epitopes in our construct, 29 were found to be antigenic in BALB/c, and for MHCII, 6 epitopes were predicted (percentile rank 1), of which WTAVVAIEPHVNPVD, IHSEFYIIPRSQESK, and TAVVAIEPHVNPVDR were antigenic epitopes.

**Table 1:** *In silico* analysis of predicted BALB/c MHC class Ι.

Allele	Peptide	Start	End	Percentile rank	Antigenicity
H-2-Ld	LPSTLRWFF	252	260	0.02	0.37
H-2-Dd	**RGPLDLTII**	267	275	0.02	**1.20**
H-2-Kd	**NYNHSDEYL**	364	372	0.07	**0.47**
H-2-Kd	**EYLSFSCYL**	370	378	0.01	**1.53**
H-2-Dd	**HGLPSTLRW**	250	258	0.11	**0.42**
H-2-Dd	FTPPNDYWI	7	15	0.13	0.05
H-2-Dd	**ISITIHSEF**	129	137	0.16	**1.26**
H-2-Ld	PPHGLPSTL	248	256	0.17	0.02
H-2-Ld	**GPLDLTIII**	268	276	0.19	**1.54**
H-2-Dd	**RNDSNKWKF**	64	72	0.25	**1.04**
H-2-Kd	RWFFNLFQL	257	265	0.29	0.33
H-2-Kd	KYGGRVWTF	101	109	0.32	0.23
H-2-Ld	**NPVDRQYTI**	46	54	0.36	**0.93**
H-2-Ld	**ISITIHSEF**	129	137	0.37	**1.26**
H-2-Dd	STSNPPHGL	244	252	0.37	0.22
H-2-Dd	TPPNDYWIL	8	16	0.4	0.04
H-2-Dd	**SNKWKFLEM**	67	75	0.43	**0.67**
H-2-Ld	TPPNDYWIL	8	16	0.47	0.04
H-2-Dd	**KEYTFPITL**	234	242	0.47	**1.29**
H-2-Dd	**STLRWFFNL**	254	262	0.51	**0.71**
H-2-Dd	**TSDTRLVGI**	91	99	0.51	**0.78**
H-2-Dd	**NSNNKEYTF**	230	238	0.56	**0.97**
H-2-Dd	**RTGNIQIRL**	323	331	0.57	**1.84**
H-2-Ld	**MAWFTPVGL**	285	293	0.6	**1.31**
H-2-Ld	**KEYTFPITL**	234	242	0.6	**1.29**
H-2-Kd	SYLYAVSGA	335	343	0.62	0.39
H-2-Kd	KFGNSIVKM	208	216	0.63	0.09
H-2-Kd	KEYTFPITL	234	242	0.63	**1.23**
H-2-Dd	**IIVSKTSLW**	186	194	0.64	**0.52**
H-2-Dd	**VGLAVDTPW**	291	299	0.7	**0.53**
H-2-Dd	YNRDIIIRF	199	207	0.72	0.39
H-2-Kd	**IRLPWYSYL**	329	337	0.75	**0.71**
H-2-Kd	**TFGLVSIQI**	354	362	0.79	**2.09**
H-2-Dd	**LSIDYKTAL**	306	314	0.8	**1.47**
H-2-Dd	**KTALGAVRF**	311	319	0.83	**0.89**
H-2-Kd	**WFTPVGLAV**	287	295	0.84	**1.40**
H-2-Dd	**SSTANLNNI**	121	129	0.84	**0.68**
H-2-Dd	RTLTSDTRL	88	96	0.88	0.21
H-2-Kd	**IRFKFGNSI**	205	213	0.92	**1.14**
H-2-Kd	**YRGPLDLTI**	266	274	0.93	**1.01**
H-2-Ld	LPWYSYLYA	331	339	0.96	0.35
H-2-Kd	**RFKFGNSIV**	206	214	0.98	**0.46**

∗Antigenicity scores ≥0.4 are shown in bold.

Using the IEDB MHC prediction tool, the truncated VP8-VP1 protein sequence was compared to human MHC class I and II with well-known HLAs in the Persian population. Table 2, Table 3 illustrate, respectively, the binding peptides for MHC class I (29 epitopes) and II (29 epitopes), some of which interact with several HLAs. Finally, neither the class I nor the class II HLA binders were poisonous nor allergenic.

**Table 2:** Predicted MHC class Ι epitopes in human.

Allele	Peptide	Start	End	Percentile rank	Toxicity	Antigenicity	Allergenicity
HLA-A^a^02:06	AIEPHVNPV	40	48	0.21	Non-Toxin	1.0847	Non-Allergen
HLA-A^a^02:01				0.45			
HLA-A^a^01:01	ANYNHSDEY	363	371	0.6	Non-Toxin	0.5623	Non-Allergen
HLA-A^a^26:01				0.85			
HLA-A^a^02:06	AVVAIEPHV	37	45	0.08	Non-Toxin	0.7941	Non-Allergen
HLA-A^a^02:01				0.33			
HLA-A^a^26:01	DIIVSKTSL	185	193	0.5	Non-Toxin	0.8737	Non-Allergen
HLA-B^a^35:03				0.58			
HLA-B^a^51:01	DSTFGLVSI	352	360	0.26	Non-Toxin	1.9570	Non-Allergen
HLA-A^a^11:01	ESALSIDYK	303	311	0.88	Non-Toxin	1.8557	Non-Allergen
HLA-A^a^24:02	EYLSFSCYL	370	378	0.27	Non-Toxin	1.5388	Non-Allergen
HLA-B^a^51:01	GPLDLTIII	268	276	0.21	Non-Toxin	1.5477	Non-Allergen
HLA-B^a^35:03				0.31			
HLA-B^a^57:01	HGLPSTLRW	250	258	0.02	Non-Toxin	0.4250	Non-Allergen
HLA-B^a^50:01	IDYKTALGA	308	316	0.59	Non-Toxin	1.3147	Non-Allergen
HLA-A^a^32:01		186	194	0.06	Non-Toxin	0.5202	Non-Allergen
HLA-B^a^57:01	IIVSKTSLW			0.12			
HLA-A^a^26:01				0.82			
HLA-A^a^24:02				1			
HLA-B^a^27:02	IRFKFGNSI	205	213	0.14	Non-Toxin	1.1449	Non-Allergen
HLA-A^a^32:01	KTALGAVRF	311	319	0.03	Non-Toxin	0.8993	Non-Allergen
HLA-B^a^57:01				0.13			
HLA-A^a^24:02				0.82			
HLA-A^a^01:01	KTDSTFGLV	350	358	0.5	Non-Toxin	1.6797	Non-Allergen
HLA-A^a^02:06				0.94			
HLA-B^a^35:01	LGDKTDSTF	347	355	0.94	Non-Toxin	1.3270	Non-Allergen
HLA-A^a^11:01	LSSRSIQYK	170	178	0.18	Non-Toxin	1.1724	Non-Allergen
HLA-A^a^03:01				0.41			
HLA-A^a^26:01		357	365	0.17			Non-Allergen
HLA-B^a^35:01	LVSIQIANY			0.58	Non-Toxin	1.4207	
HLA-A^a^01:01				0.62			
HLA-A^a^02:01		125	133	0.23			Non-Allergen
HLA-A^a^32:01	NLNNISITI			0.53	Non-Toxin	1.7404	
HLA-A^a^02:06				0.7			
HLA-B^a^57:01		230	238	0.64	Non-Toxin	0.9777	Non-Allergen
HLA-B^a^35:01	NSNNKEYTF			0.66			
HLA-A^a^32:01				0.73			
HLA-A^a^26:01	NSNTNGVVY	18	26	0.54	Non-Toxin	0.3576	Non-Allergen
HLA-B^a^51:01	PPIQNTRNV	157	165	0.58	Non-Toxin	1.5373	Non-Allergen
HLA-A^a^32:01	RDIIIRFKF	201	209	0.99	Non-Toxin	1.5910	Non-Allergen
HLA-A^a^32:01	RTGNIQIRL	323	331	0.13	Non-Toxin	1.8437	Non-Allergen
HLA-B^a^57:01				0.56			
HLA-A^a^11:01	SSRSIQYKR	171	179	0.58	Non-Toxin	0.8972	Non-Allergen
HLA-B^a^51:01	TANLNNISI	123	131	0.25	Non-Toxin	1.2061	Non-Allergen
HLA-B^a^35:03				0.37			
HLA-B^a^50:01	TEQSEFYFP	381	389	0.41	Non-Toxin	0.6569	Non-Allergen
HLA-A^a^24:02	TFGLVSIQI	354	362	0.66	Non-Toxin	2.0992	Non-Allergen
HLA-B^a^35:03	TPWVEKESA	297	305	0.6	Non-Toxin	0.4216	Non-Allergen
HLA-B^a^35:01				0.95			
HLA-B^a^50:01	VEKESALSI	300	308	0.08	Non-Toxin	1.0647	Non-Allergen
HLA-A^a^02:06	YINNGLPPI	151	159	0.11	Non-Toxin	0.9097	Non-Allergen
HLA-A^a^02:01				0.18			

a Antigenicity scores ≥0.4 are shown in bold.

**Table 3:** Predicted MHC class IΙ binders in human.

Allele	Peptide	Start	End	Percentile rank	Toxicity	Allergenicity	Antigenicity
HLA-DRB1^a^11:53	AYTFNSNNKEYTFPI	226	240	0.58	Non-Toxin	NON ALEERHEN	0.88
HLA-DRB1^a^03:27	CNEYINNGLPPIQNT	148	162	0.6	Non-Toxin	NON ALEERHEN	0.64
HLA-DRB1^a^13:62	DTRLVGILKYGGRVW	93	107	0.84	Non-Toxin	NON ALEERHEN	0.14
HLA-DRB1^a^03:40	EKESALSIDYKTALG	301	315	0.96	Non-Toxin	NON ALEERHEN	1.36
HLA-DRB1^a^03:05	EMQYNRDIIIRFKFG	196	210	0.81	Non-Toxin	NON ALEERHEN	0.65
HLA-DRB1^a^11:17	EPHVNPVDRQYTIFG	42	56	1	Non-Toxin	NON ALEERHEN	1.25
HLA-DRB1^a^03:19	FYNRRTLTSDTRLVG	84	98	0.96	Non-Toxin	NON ALEERHEN	0.46
HLA-DRB1^a^04:66	HSEFYIIPRSQESKC	134	148	0.93	Non-Toxin	NON ALEERHEN	0.58
HLA-DRB1^a^04:89	IQYKRAQVNEDIIVS	175	189	0.5	Non-Toxin	NON ALEERHEN	0.42
HLA-DRB1^a^03:27	KCNEYINNGLPPIQN	147	161	0.83	Non-Toxin	NON ALEERHEN	0.63
HLA-DRB1^a^03:38	KESALSIDYKTALGA	302	316	0.98	Non-Toxin	NON ALEERHEN	1.34
HLA-DRB1^a^07:06	LDLTIIITGATDVDG	270	284	0.52	Non-Toxin	NON ALEERHEN	0.61
HLA-DRB1^a^03:27	NEYINNGLPPIQNTR	149	163	0.58	Non-Toxin	NON ALEERHEN	0.98
HLA-DRB1^a^04:51	NGVVYESTNNSDFWT	22	36	1	Non-Toxin	NON ALEERHEN	−0.05
HLA-DRB1^a^04:09	NTNGVVYESTNNSDF	20	34	0.9	Non-Toxin	NON ALEERHEN	0.38
HLA-DRB1^a^07:11	PLDLTIIITGATDVD	269	283	0.98	Non-Toxin	NON ALEERHEN	1.2
HLA-DRB1^a^04:27	RSIQYKRAQVNEDII	173	187	0.99	Non-Toxin	NON ALEERHEN	0.14
HLA-DRB1^a^11:27	SDTRLVGILKYGGRV	92	106	0.99	Non-Toxin	NON ALEERHEN	0.44
HLA-DRB1^a^11:81	SEFYIIPRSQESKCN	135	149	1	Non-Toxin	NON ALEERHEN	0.46
HLA-DRB1^a^13:10	SIQYKRAQVNEDIIV	174	188	1	Non-Toxin	NON ALEERHEN	0.46
HLA-DRB1^a^04:82	SRSIQYKRAQVNEDI	172	186	0.77	Non-Toxin	NON ALEERHEN	0.28
HLA-DRB1^a^04:14	SSQNEFYNRRTLTSD	79	93	0.78	Non-Toxin	NON ALEERHEN	0.29
HLA-DRB1^a^04:87	SSRSIQYKRAQVNED	171	185	0.98	Non-Toxin	NON ALEERHEN	0.4
HLA-DRB1^a^04:07	TNGVVYESTNNSDFW	21	35	1	Non-Toxin	NON ALEERHEN	0.14
HLA-DRB1^a^11:54	TSDTRLVGILKYGGR	91	105	0.9	Non-Toxin	NON ALEERHEN	0.49
HLA-DRB1^a^13:32	VVAIEPHVNPVDRQY	38	52	0.98	Non-Toxin	NON ALEERHEN	1.22
HLA-DRB1^a^04:07	WSAAYTFNSNNKEYT	223	237	0.95	Non-Toxin	NON ALEERHEN	0.61

a Antigenicity scores ≥0.4 are shown in bold.

**Fig. 2:**
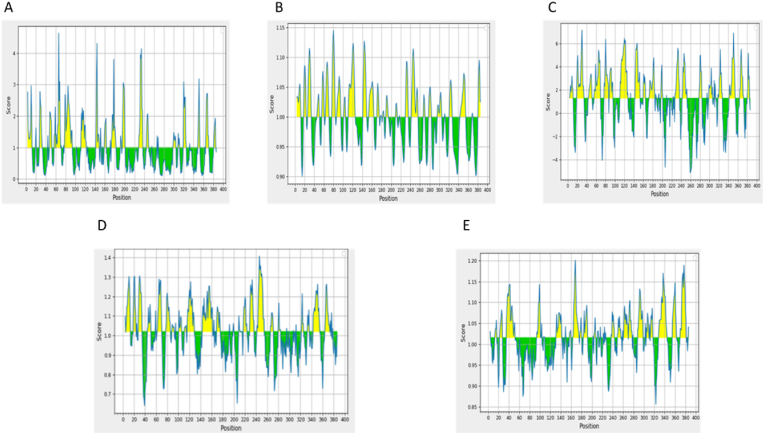
(a) Emini surface accessibility, (b) Karplus and Schulz flexibility, (c) Parker hydrophilicity, (d) Chou and Fasman beta-turn prediction, and (e) Kolaskr and tongaonkar antigenicity where X- and Y-axis represent the sequence position and respective scores. The line represents the average values of the respective calculated scores and the yellow regions above the average are considered as good.

B cells produce antibodies as part of humoral immunity. Long-lasting immunity is provided through B-cell receptors' recognition of B-cell epitopes. The ABCpred server discovered 26 epitopes with a score of 0.5 or higher and a length of 16 mer, which the BepiPred tool then verified. Their hydrophilicity, surface accessibility, antigenicity, flexibility, and beta-turn were used to describe them. The mean scores for Emini surface accessibility, Karplus and Schulz flexibility, Parker hydrophilicity, and Kolaskr and tongaonkar antigenicity were determined to be 1.000 (Fig. 2a), 1.000 (Figs. 2b), 1.296 (Fig. 2c), and 1.016 (Fig. 2e), respectively. At the same time, Chou and Fasman's method was utilized to forecast beta-becomes since beta turns hydrophilic and aids in triggering the immunological response (Rose et al., 1985). The mean, lowest, and highest beta-turn scores were 1.020, 0.641, and 1.407, respectively (Fig. 2d). The Ellipro server produced six conformational and thirteen linear B-cell epitopes (Table 4, Table 5). PyMOL examined the mapped epitopes.

**Table 4:** Discontinuous B-cell epitopes predicted by Ellipro for model.

No	Residues	Number of residues	Score	3D model
1	A: Y177, A: K178, A: R179, A: A180, A: Q181, A: V182, A: N183, A: E184	8	0.976	
2	A: D185, A: I186, A: I187, A: V188, A: S189, A: K190, A: T191, A: S192, A: L193	9	0.97	
3	A: W194, A: K195, A: E196, A:M197, A: Q198, A: Y199, A: N200, A: L378, A: S379, A: V380, A: T381, A: E382, A: Q383, A: S384, A: E385, A: Y387	16	0.827	
4	A:N11, A:D12, A:S19, A:N20, A:T21, A:N22, A:G23, A:T29, A:N30, A:N31, A:S32, A:D33, A:F34, A:W35, A:A40, A:I41, A:E42, A:P43, A:H44, A:V45, A:N46, A:P47, A:V48, A:D49, A:R50, A:Q51, A:Y52, A:T53, A:I54, A:F55, A:G56, A:E57, A:S58, A:K59, A:Q60, A:F61, A:N62, A:V63, A:R64, A:N65, A:D66, A:S67, A:N68, A:K69, A:W70, A:K71, A:F72, A:F76, A:R77, A:S78, A:S79, A:S80, A:Q81, A:N82, A:E83, A:F84, A:Y85, A:R87, A:R88, A:T89, A:L90, A:T91, A:S92, A:D93, A:T94, A:R95, A:L96, A:Y102, A:G103, A:G104, A:R105, A:W107, A:H110, A:G111, A:E112, A:T113, A:P114, A:R115, A:A116, A:T117, A:T118, A:D119, A:S120, A:S121, A:S122, A:T123, A:A124, A:N125, A:L126, A:N127, A:N128, A:I129, A:S146, A:N149, A:E150, A:N153, A:N154, A:G155	98	0.681	
5	A:R201, A:D202, A:I203, A:I204, A:R206, A:K208, A:Y236, A:T237, A:F238, A:P239, A:D271, A:L272, A:T273, A:I275, A:I276, A:T280, A:D281, A:V282, A:D283, A:G284, A:M285, A:A286, A:W287, A:F288, A:T289, A:P290, A:V291, A:T321, A:R322, A:T324, A:G325, A:N326, A:I327, A:Q328, A:I329, A:R330, A:L331, A:P332, A:W333, A:Y334, A:S335, A:Y336, A:L337, A:A339, A:L344, A:D345, A:G346, A:L347, A:G348, A:D349, A:K350, A:T351, A:D352, A:S353, A:T354, A:F355, A:G356, A:N364, A:N366, A:H367, A:S368, A:D369, A:E370, A:Y371, A:S373, A:F374, A:S375, A:C376, A:Y377	69	0.601	
6	A: S171, A: S172, A: R173, A: S174, A: I175, A: Q176, A: Q264, A: L265, A: Y266, A: T297, A: P298, A: W299, A: V300, A: E301, A: K302, A: A305, A:L306, A:S307, A:I308, A:D309, A:Y310, A:K311, A:T312, A:A313, A:L314, A:G315	26	0.57

**Table 5:** Population coverage of construct.

Population	MHC I PPC	Average of Epitope Hits	MHC II PPC	Average of Epitope Hits
Iran	88.37 %	42.14	94.14 %	14.6
World	91.57 %	44.57	88.58 %	12.85

### 3.2 Antigenicity, immunogenicity, population coverage, and epitope conservancy analysis

An immune-prophylactic vaccine must meet the key requirement of antigenicity. The antigenicity of the suggested vaccine construct was assessed using VaxiJen 2.0 servers to make sure that it would engage with the B and T cell receptors to trigger a sustained immune response. Due to the findings in Table 1, Table 2, Table 3, high-affinity peptides with the antigenic characteristic (percentile rank 1) are included. Table 5 contains the findings of the overall population coverage in the Persian population.

### 3.3 Primary and secondary structure analysis

The fusion protein with molecular weight and specific model, which is the theoretical isoelectric point, and the hydropathic average - indicating the solubility and hydrophobicity of the desired protein - were calculated using the ProtParam Expasy service. The fusion model has 35 positively charged (Arg + Lys) and 34 negatively charged (Asp + Glu) residues in a polypeptide with a molecular weight of 44.42 kDa and 389 amino acids. This model is also projected to be hydrophilic and dissolvable (GRAVY Great Normal of Hydropathy: −0.397). Using secondary structure analysis and disulfide connections, the SOPMA programme was utilized to predict the secondary structure of Model characteristics such as beta turns, helices, random coil contribution, and C-score. A larger ratio of random coils to extended strands is associated with an increase in the production of protein antigenic epitopes. As a result, 15.17 percent of it is a helix, and 35.48 percent of it is a sheet, which, along with the largest percentage of random coils (42.16) can produce greater antigenic epitopes. Fusion Protein Model predicts the presence of a single disulfide bridge between and that offers stability external to the cell.

### 3.4 Tertiary structure prediction and validation

**Fig. 3:**
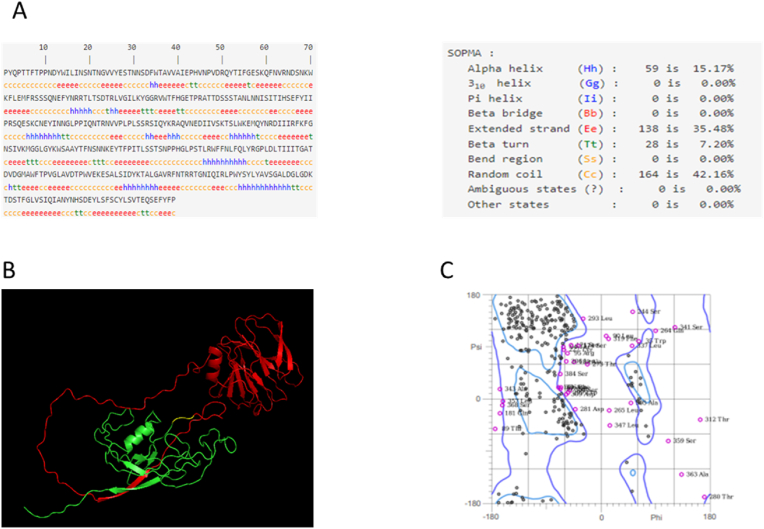
Sequence and structural analysis of Model. (A) Secondary structure by SOPMA tool, (B) Three-dimensional structure by PyMOL red: truncated vp8, yellow: linker and green: truncated vp1. (C) Ramachandran plot of the vaccine structure (70.0 % (271/387) of all residues were in favored regions. 87.3 % (338/387) of all residues were in allowed regions (http://molprobity.biochem.duke.edu).

**Fig. 4:**
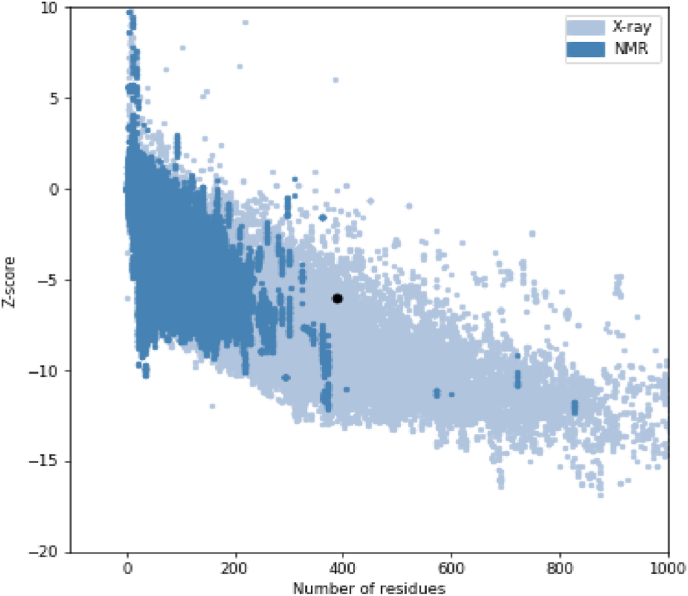
PROSA validation of 3D vaccine structure showing Z-score (−6.04).

Model's three-dimensional structure was predicted using the online I-TASSER service, which provides five top models with C-scores ranging from −5 to 2. The model with the greatest C-score is the most effective one. This value's confidence level was adequate (−1.06) for the model that was chosen. Using PyMOL, the protein picture was produced. Additionally, the Ramachandran plot demonstrated that 271/387, or 70.0 %, of all residues, were in favored areas. A total of 338/387 residues, or 87.3 %, were in permitted locations (Fig. 3). The model's 3D structure had a Z-score of 6.04 (Fig. 4).

### 3.5 Molecular docking

**Fig. 5:**
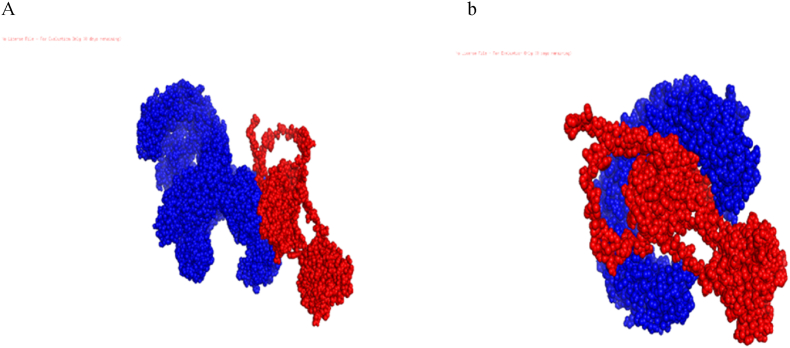
Interaction between the proposed vaccine construct and TLR3, TLR4, Insilico docking between TLR3, TLR4, and subunit vaccine construct was predicted using ClusPro v2.0 tool; the vaccine construct and the receptor are in red and blue colors, respectively.

Toll-like receptors (TLRs) are very important for recognizing pathogen-related molecular patterns and regulating the induction of immune responses in viral infections, especially hepatitis A and rotavirus. In this study, out of two Toll-like receptors (TLRs) (4 TLR3) that TLR3 recognizes viral glycoproteins and lipoproteins and activates immune cells. Activation of TLR4 leads to inflammation and secretion of inflammatory cytokines such as interleukin-6 and 5 through a MyD88-related pathway and also promotes gamma interferon secretion through a MyD88-independent pathway. On the other hand, activation of TLRs-related immune pathways could be therapeutic for hepatitis A rotavirus infections. To evaluate the candidate vaccine binding affinity with TLRs, we used the ClusPro v2.0 binding tool, which is very helpful for evaluating the induction and immune responses. For each TLR binding vaccine design, thirty anchor complexes were generated and the corresponding centers and their lowest energy score was determined. Vaccine/TLR3 and vaccine/TLR4 complexes showed the center score and the lowest energy. The final output with the lowest global energy (−1089) for the vaccine/TLR3 construct and (−1006.8) for our vaccine/TLR4 construct was considered the most favorable interaction. Interactions between vaccine candidates and TLRs were visualized using ClusPro v2.0 tool (Fig. 5).

## 4 Discussion

Highly infectious viruses like rotavirus and hepatitis A can generate seasonal epidemics and pose a serious risk to the public's health. These viruses are able to easily cross boundaries and infect a variety of organisms. As a result, it is crucial to put in place efficient preventative measures, such vaccinations. Unfortunately, the lack of a bivalent vaccination highlights the necessity for a vaccine or pharmacological intervention to manage these epidemics notwithstanding the high fatality rates and astronomical medical costs associated with these infections.^22^ In contrast to traditional vaccination procedures that depend on attenuated or inactivated viral strains, bioinformatics analysis offers a speedier and less complex process for creating recombinant vaccines. The bioinformatics method offers a quicker and more effective technique to find possible vaccine epitopes.^29^

In this study, we identified functional epitopes of a fusion protein of rotavirus and hepatitis A viruses (VP8∗-rotavirus + AAY + HAV-VP1 fusion protein), including 389 amino acids with high immunogenicity to stimulate T cells and B cells, using advances in bioinformatics tools, the availability of viral genome databases, and previous studies.

For rotavirus and hepatitis A, many vaccinations have been investigated. While full-length or truncated VP8∗ proteins and clinical studies with the p2∗- VP8 (two fused VP8∗ copies) subunit vaccine have also showed promising outcomes, recombinant VP6 proteins have been studied as vaccine candidates for rotavirus. Previous research has examined the effectiveness of Vi-VP8 (comprising many rotavirus groups) combinations in boosting the immune response with a focus on various rotavirus species. According to Mickley et al., the Vi-VP8 conjugation could induce persistent immunity and had a molecular weight of 49.50 kDa. However, Dormitzer et al. (2002) noted that this strategy may not be successful due to the notable discrepancies between the antigenic epitopes of the VP8∗ region of rotavirus A strains circulating in different regions of the world. Because the VP8∗ fragment has more genetic diversity, is more effective against a variety of rotavirus A strains, offers long-term protection from the virus, and also triggers a potent and specific immune response against rotavirus A, we chose to concentrate on it rather than the VP4 fragment. (2012) Zeller et al.^17^^,^^19^ According to earlier research and immunogenicity analyses, long-term booster doses of live, attenuated/inactivated hepatitis A vaccinations resulted in considerable seroconversion and protective protection. However, the sluggish rate of HAV proliferation in cell culture results in low yield, making the manufacture of inactivated vaccines challenging and expensive. In terms of safety, high purity, and well defined structures, recombinant subunit vaccines, which have the capacity to elicit an effective immune response, seem more sensible.^44^^,^^51^

Recombinant proteins can be used to identify particular epitopes, minimize the risk of adverse responses, and examine prospective epitopes in proteins, which provides the possibility of producing vaccines with high ability to fight effectively against diseases caused by viral agents^33^ The discovered epitopes in this work have the capacity to trigger humoral immune reactions from memory B cells and to trigger particular immunological reactions against rotavirus and hepatitis A. They can also boost CTL and Th cell activity and produce CTL-mediated responses, which are crucial for controlling viral infection. The promotion of effective CD8^+^ CTL responses and durable antibody immunological responses through the induction of T-cell-mediated immunity lowers the incidence of illness. Because these epitopes may elicit a potent and focused immune response against rotavirus and hepatitis A, their application in the creation of vaccines holds promise for stopping the spread of these viral diseases.^31^ Analysis of the intended structure in revealed the expected epitopes' affinity for mouse MHC-I and II molecules. It was discovered that we have a large number of epitopes with the strongest affinity for binding to mouse MHC-I alleles, and molecular binding study revealed the Mouse alleles with the highest mutual similarity score were H-2-Ld, H-2-Dd, and H-Dd2. The antigenicity of the VP8∗ region's antigenic epitopes may be impacted by various amino acid variations in their exposed areas. The degree of T cell immunogenicity is significantly influenced by the T cell epitopes' ability to bind to the MHC. Since epitope binding to MHC molecules is necessary for antigen presentation to T cells, it is crucial to find epitopes that interact with MHC I and MHC II polymorphic alleles that have high population coverage scores.

Similar research was done on rotavirus from vp7 and vp8, however only three CTL epitopes and four HTL epitopes were found in B cells. This emphasizes the significance of utilizing bioinformatics methods to locate possible vaccine epitopes since they can assist locate certain epitopes that might trigger robust and focused immune responses against viral infections. It is crucial to identify strong epitopes for the rotavirus and hepatitis A viruses in order to create vaccinations that are effective against these viral illnesses. Muhammad Osman as well as others. The strongest epitopes in this investigation were determined to be 9 CTL and HTL epitopes from 389 amino acids with the greatest binding affinities for various MHC molecules and high immunity and protection ratings. For instance, the Pol (215–258) epitope has the maximum binding affinity for five HLA supertypes and 16 common HLA-I alleles. For a number of HLA-B and HLA-A alleles, the epitope also demonstrated strong binding affinity. The secondary and tertiary structures of the vaccine need epitopes connected to the TCR/BCR by the linker sequences AAY and EAAAK. It was discovered that the vaccine's structure comprises of 40 % coiled-coil, 42 % beta sheets, and 16 % alpha helix.^25^

The study looked at the physical and chemical characteristics of the vaccine structure, and it was discovered that it had an aliphatic index, a possible hydrophobicity, and a molecular weight of 44.42 kDa. The peptide's total hydropathic average (GRAVY) was 0.381, which designates it as hydrophilic. These traits show that the vaccine protein is thermally stable and appropriate for the rotavirus and hepatitis A vaccines. Alpha helices, extended strands, beta twists, and random helical structures were discovered in the vaccine's secondary and tertiary structures, which were also examined. The Ramachandran diagram's predictions indicated that the three-dimensional structure would have advantageous properties. The vaccine's structural flaws and potential modelling issues were corrected using the ProSA programme, which was also used to determine the structure's overall quality score.

The linear epitope prediction was assessed by the amino surface accessibility, Kulascar and Tongaonkar antigenicity test, and Parker hydrophilicity test in order to establish that the vaccine binds to B cells. Epitopes over the threshold were anticipated, and it was discovered that the B cell's surface and epitopes were “hydrophilic”. IEDB techniques were used to analyze the target sequence's expected MHC I and II binding to T cells, and it was discovered that T cells are projected to interact with MHC I alleles with an IC_50_ = 100. However, the study also discovered that certain amino acid variations occur in the exposed areas of the VP8∗ region's antigenic epitopes, and these variations may have an impact on antigenicity. Therefore, more research and optimization may be needed to guarantee the vaccine's effectiveness. Assessment, which indicates that this model. The structural analysis of this model revealed that the model that received the highest score (0.994) contained a protein with 389 amino acids and 42.16 % random coils. Additionally, the Ramachandran diagram revealed that there were 91.7 % non-toxic amino acids in the protein, with a high population coverage, particularly in Iran and Europe. Performance evaluation of protein-protein binding affinity, protein fusion candidate vaccine with mediators of innate immune stimulation actually interacts with rotavirus VP1-HAV ^52^ and rotavirus, VP8^53^ genes with Toll-like receptors (TLRs) for pathogen recognition (eg, TLR3, TLR4) showed that the interaction between the fusion protein can lead to the induction of the release of inflammatory cytokines such as interleukin 6 and 5, as well as promoting the release of gamma interferon and creating an effective innate immune response, which indicates the good performance of the fusion protein as a vaccine candidate against the spread of hepatitis A virus.^54^ that the accuracy of the desired study requires experimental and clinical validation. Immunity against diseases is developed by vaccination, which also provides antigenic agents to activate the immune system. On the other hand, immunoinformatic approaches are a very effective tool for designing and studying algorithms to reduce time and cost, as well as the trend of increasing the propagation chain of rotavirus infection and the prevalence of hepatitis A in children. At the moment, there is no effective drug and the time-consuming process of treatment and drug. And the only way to regulate the poor economic conditions in emerging and third-world nations is by offering a possible vaccination. We created a protein fusion as a possible dual vaccination candidate using bioinformatics tools and methodologies.^26^ The chosen recombinant protein is next tested *in vitro* and *in vivo* to see how strongly it can elicit immunological responses. It may be utilized efficiently to create vaccines quickly and affordably. Although the Insilco results show that the vaccination is efficacious, laboratory testing and animal model studies need be done to evaluate the vaccine's therapeutic efficacy. Additionally, the use of anticipated peptides may result in the creation of native variant DNA vaccines. For *in vitro* and *in vivo* investigations, particular attention should be paid to residues that exhibit minimal variability, stimulate B-cell immunity, and have a high affinity to alleles. Additionally, the variety of projected epitopes will rise as a result of the research of molecular epidemiology in many worlds logical systems. As a result, we advise conducting further fieldwork, particularly in nations where there is little knowledge about the rotavirus and hepatitis A strains that are in circulation. It will then be put to use in research.

## 5 Conclusion

We designed a fusion protein using bioinformatics tools and methods as a viable recombinant vaccination candidate. Hepatitis A virus VP1 and rotavirus VP8 protein epitopes are present in this vaccination. The chosen recombinant protein can substantially elicit immunological responses and may help in the development of an efficient vaccine to decrease the prevalence of rotavirus and hepatitis A in various populations.

## CRediT authorship contribution statement

**Hassan Yarmohammadi:** Writing – original draft. **Abbas Akhavan Sepahi:** Formal analysis. **Mojtaba Hamidi-fard:** Data curation. **Mohammadreza Aghasadeghi:** Validation. **Golnaz Bahramali:** Writing – review & editing.

## Declaration of competing interest

The authors declare that they have no known competing financial interests or personal relationships that could have appeared to influence the work reported in this paper.

## Data Availability

The data that has been used is confidential.
